# Health Benefits of Indoor Cycling: A Systematic Review

**DOI:** 10.3390/medicina55080452

**Published:** 2019-08-08

**Authors:** Manuel Chavarrias, Jorge Carlos-Vivas, Daniel Collado-Mateo, Jorge Pérez-Gómez

**Affiliations:** 1 Faculty of Sport Science, University of Extremadura, 10003 Cáceres, Spain; 2 UCAM Research Center for High Performance Sport, Catholic University of Murcia, 30107 Murcia, Spain

**Keywords:** aerobic capacity, blood pressure, body mass index, indoor bicycle, spinning exercise

## Abstract

*Background and Objectives:* Indoor cycling is one of the most practiced activities in fitness centers for most people regardless of their physical conditioning level. Several studies have analyzed the effect of indoor cycling on several parameters related to health, such as maximal oxygen consumption, blood pressure, body composition, as well as biochemical markers such as HDL or LDL. However, no study has synthesized all health benefits associated with the indoor cycling practice in the form of a systematic review and established guidelines or recommendations. Therefore, the aim of this manuscript was to conduct a systematic review of published studies about the benefits of indoor cycling training and to establish recommendations for coaches, researchers, and practitioners. *Materials and Methods:* The PRISMA guidelines were followed to conduct the current systematic review. A systematic search was performed to retrieve relevant published articles until January 2019 using the following keywords: ‘indoor cycling’, ‘indoor bicycle’, and ‘spinning exercise’. Information about participants, intervention, comparisons, outcomes, and study design (PICOS) was extracted. *Results:* A total of 300 studies were initially identified. After the revision process, 13 of them were included. The total sample size of the studies was 372 (306 women). Results revealed that indoor cycling may improve aerobic capacity, blood pressure, lipid profile, and body composition. These enhancements may be achieved as standalone intervention or combined with other physical exercises or diet. *Conclusions:* The combination of indoor cycling and diet is recommended to improve the lipid profile, lose weight, and reduce blood pressure. Furthermore, indoor cycling alone may also enhance aerobic capacity. Given the lack of randomized controlled trials, these conclusions should be taken with caution.

## 1. Introduction

Indoor cycling (IC), also known as spinning, is a physical activity offered in most gyms. Participants of different ages, body mass indices (BMI), and physical fitness cycle on modified stationary bikes following the music rhythm and the instructions of the IC trainer. The choreography of the music plays an important role in IC because it may modify the participant’s motivation and the intensity of the exercise [1].

The IC coach controls intensity to reach in each music track and the participants have to adjust the tension in the steering wheel. Indicators of training intensity, such as heart rate (HR) and rating of perceived exertion, can allow participants to measure their results and control their performance within safe ranges, avoiding over exertion and maximizing the benefits of their time and effort of training [2].

The intensity of IC activity is strongly associated with changes in position, music rhythm, cadence, and revolutions per minute. Therefore, the IC trainers can select the intensity of a training session depending on the fitness level of the participants. In this way, it is the instructor who decides and monitors the workload of the IC session [3].

IC is a fitness activity characterized by steps of workout with variable intensity and a high/moderate involvement of the cardiovascular system as well as the skeletal muscles [3,4]. Cycling serves both as a method of physical conditioning and as a method of rehabilitation through exercise. Due to the use of a reciprocal vertical movement similar to walking, it equally plays an important role in physical conditioning and rehabilitation centers [5]. 

Some risk factors at the muscle level during cycling are fatigue and decreased muscle control, poor technique, or lack of conditioning [6]. Several studies have been focused on IC measuring fatigue [7], adaptations [8], and cardiorespiratory and metabolic response [9]. However, to our knowledge, there is no systematic review of all health benefits that IC practice can produce on the participants. Therefore, the purpose of this study was to review and identify which kind of health benefits have been described in the research associated with IC practice.

## 2. Materials and Methods

### 2.1. Search Strategy

The PRISMA (Preferred Reporting Items for Systematic reviews and Meta-Analyses) [10] guideline has been followed to conduct the present systematic review.

A systematic review of the studies was conducted up to January 2019. The search was performed by the authors ‘M.C.’ and ‘J.P.-G.’ in the following electronic databases: Pubmed (Medline), Web of Science (including the Current Contents Connect, the Korean Journal Database, Medline, SciELO and the Russian Science Citation Index) and the Physioterapy Evidence Database. The relevant articles were searched using the terms ‘indoor cycling’, ‘indoor bicycle’, and ‘spinning exercise’. The filters used in PubMed to include the articles were: published in English or Spanish languages. In Web of Science, patents, abstracts, meetings, books, reviews, letters, and editorials were excluded. Again, only articles written in Spanish or English were included. Furthermore, articles from the following areas were excluded: energy fuels, mathematics, business economics, meteorology, atmospheric sciences, toxicology, architecture, construction building technology, art, chemistry, and information science/library science.

The inclusion criteria for the articles selected were (1) those that measure any health-related physiological or body composition parameter before and after IC intervention, (2) original research, and (3) written in English or Spanish. 

The exclusion criteria for articles were set as follows: (1) not measuring the effect of IC on any body composition or physiological health parameters; (2) related to pathologies or injuries such as rhabdomyolysis, ischemic heart disease, compartment syndrome, fractures or thrombosis; (3) cycling performance and validation of measuring devices or substances for performance; (4) no numerical data reported; and (5) focused on acute effects.

To follow the PRISMA guidelines, data extraction was conducted by the authors ‘M.C’ and ‘J.P-G.’ taking into account the PICOS approach, which includes: participants, intervention, comparisons, outcomes, and study design (PICOS) [10].

### 2.2. Participants

A total of 66 men and 306 women participated in the 13 studies included in this systematic review (Table 1). Regarding age, one study was conducted with girls aged around 13 [11], four studies were carried out with young adults aged <40, and the remaining eight studies included participants with mean ages between 42.9 [12] and around 60 [13]. Most participants were healthy subjects, but some studies were conducted with other special populations, such as those with fibromyalgia, metabolic syndrome, diabetes, or who are overweight. Mean BMI was always lower than 30, except in Tsai et al. [14], and Mensberg, et al. [15], that involved participants with diabetes or metabolic syndrome and a mean BMI slightly higher than 30.4 kg/m^2^ [14,15].

### 2.3. Interventions

Table 2 summarizes the exercise interventions of the studies included in this systematic review. Duration of the interventions ranged from 8 [12] to 24 weeks [13,16]. Furthermore, frequency and duration of the sessions were very heterogeneous. In this regard, weekly sessions oscillated between 2, 5, and 6, while duration of the sessions varied from 30 min to 100 min. The type of the interventions was also very different among studies, with nine studies conducting IC only and three studies assessing the effects of the combination of IC plus other activities such as treadmill, strength training, body pump (BP), and body balance (BB).

### 2.4. Quality of the Evidence

The GRADE approach [17,18] was used to evaluate the quality of the evidence of the included studies. It involves a scale from ‘very low’ to ‘high’. In the current study, the quality of the evidence began at the low level since there were studies with no randomization and without control group. Furthermore, the quality of the evidence was downgraded given the heterogeneity of the results and the protocols of IC interventions. Therefore, the quality of the evidence was ‘very low’, which means that “We have very little confidence in the effect estimate: The true effect is likely to be substantially different from the estimate of effect” [17]. 

## 3. Results

### 3.1. Search and Selection of Publications

**Figure 1:**
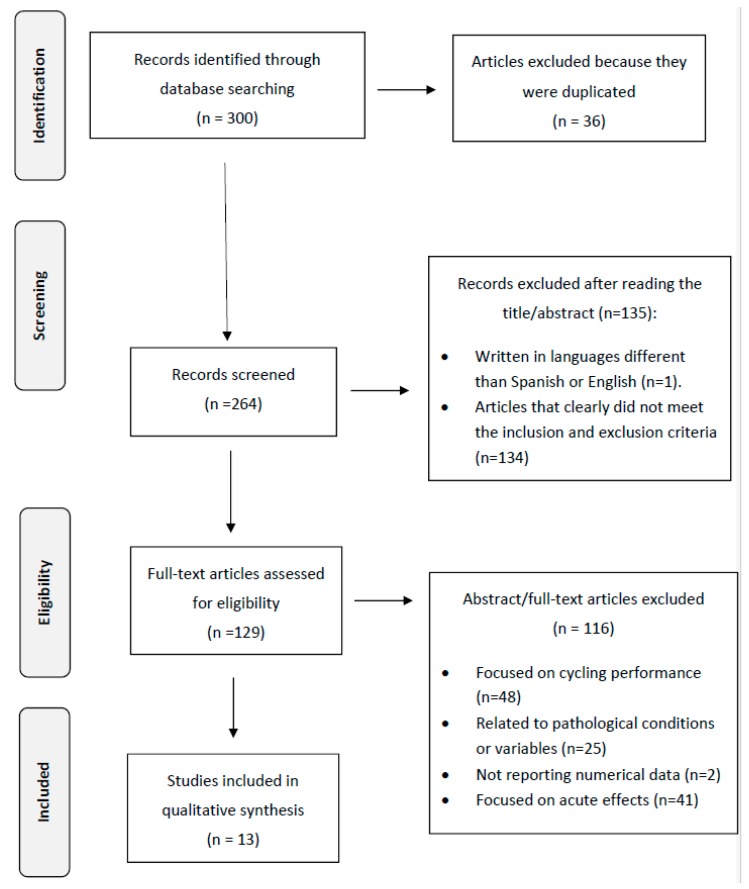
Flow diagram for selection of studies according to PRISMA guidelines.

Initially, 246 studies were identified in the Pubmed database, 50 in Web of Science, and 4 in the Physiotherapy Evidence Database. The abstracts were read for relevance, if any doubt persisted about the relevance for the aim of the current study then the full text was read. Of the total 300 articles, 36 were excluded because they were duplicated (Figure 1). Following the exclusion and inclusion criteria, the other reasons for the exclusion were: written in languages different than Spanish and English (n = 1); clearly not related to the topic (n = 134), such as the studies by Du et al. [19], Kwon et al. [20], or Velthuis et al. [21]; related to cycle performance or to other variables assessed during cycling (n = 48), such as the evaluation of the methods of adjusting saddle height [22], the effects of tramadol on physical performance [23] or heat acclimation [24]; assessing only acute effects (n = 41), such as the studies by Barbado et al. [25], Luszczyk et al. [26], or Rendos et al. [27]; related to pathologies (n = 25), such as rhabdomyolysis [28], open ankle fracture [29], or thigh compartment syndrome [30]; and without numerical data (n = 2), such as Nair et al. [31] and Shafer [32]. Finally, 13 articles were included in this systematic review.

### 3.2. Outcome Measures

The outcome measures included in the current systematic review were those that were evaluated by at least 3 of the 13 articles. These variables were: maximal oxygen consumption (VO_2max_), serum lipids (including triglycerides, total cholesterol, high density lipoproteins, and low-density lipoproteins), blood pressure (both systolic and diastolic), body mass, percentage of body fat, and lean body mass.

### 3.3. Maximal Oxygen Consumption (VO_2max_)

There were six studies that measured VO_2max_ involving 135 participants, most of them women (Table 3). There were significant within-group improvements in all studies, but no between-group difference was reported. In this regard, three of the six studies did not have a control group, and the other three compared the effects between two IC programs in different populations [33], compared the effects of 12 weeks’ intervention in women with and without fibromyalgia and observed a within group improvement in healthy subjects but not in women with chronic pain. Unexpectedly, they observed a higher %HRmax and a slightly lower perception of effort in the pathological group compared with the control group. The other two studies with a comparison group evaluated the differences of the same physical exercise program in premenopausal and postmenopausal women [34], and also the differences between adding liraglutide, which is a medication used in type 2 diabetes patients that may positively affect cardiovascular variables and reduce weight [35], or a placebo to the physical exercise intervention.

In general, there was an agreement among all studies since all of them reported significant improvements after different types of IC exercise programs.

### 3.4. Lipid Profile

The effects of the IC intervention on serum lipids were evaluated in six studies. The variables commonly reported were triglycerides (Table 4a), total cholesterol (Table 4a), and the two types of lipoproteins (Table 4b): high density lipoproteins (HDL) and low-density lipoproteins (LDL). A total of 177 participants were included in the 6 studies reporting changes in any of the serum lipids variables. Of these, 120 were women and 57 were men.

Regarding the effects in the levels of triglycerides, there were significant within-group differences in the studies by Valle, et al. [36] and Verrusio, Andreozzi, Renzi, Martinez, Longo, Musumeci, and Cacciafesta [13]. Other three studies did not observe any significant change, while in the article by Yoon et al. this variable was not included [11]. Thus only the intervention involving physical therapy and diet achieved significant improvements in people with metabolic syndrome [13], while in the study by Valle, Mello, Fortes Mde, Dantas, and Mattos [36] all three intervention groups (IC exercise, diet, and the combination of the diet and IC exercise) significantly reduced the triglyceride levels in healthy subjects.

Regarding the benefits on total cholesterol levels, three of the studies reported significant within-group differences. In the study by [11], between-group differences were observed at baseline. The comparison group performed bicycle exercise achieving a reduction lower than 5%, while the IC group reduced their triglyceride levels a 6.56%, thus both groups observed similar benefits. Two of the studies did not observe any significant changes in the total cholesterol levels after a program involving IC, strength training and placebo or liraglutide [15] and after IC exercise at home. Interestingly, these two studies included patients with diabetes or metabolic syndrome. The reduction in triglyceride levels was proportional to the initial levels, so that in the study by Verrusio, Andreozzi, Renzi, Martinez, Longo, Musumeci, and Cacciafesta [13] the reduction was around 30 mg/dl with baseline levels higher than 240 (reduction around 12.5%), and in the rest of the studies the reduction was around 10 mg/dl with baseline values between 160 and 180 (reduction around 6%).

Lastly, with regards to HDL and LDL levels, one study [36] observed a between group increment in HDL in favors of the IC groups, with increments around 8%, while the non-exercise groups (both diet and control groups) observed non-significant changes lower than 2%. That study was also the only one that observed a significant reduction in LDL levels in all groups, except in the control group (IC, diet and the combination of IC and diet). No study reported significant between-group differences in LDL levels. A significant increment in HDL levels was also observed in the study by Kyrolainen, et al. [37], which evaluated the effects of IC and strength training combination in overweight and normoweight young female adults. 

### 3.5. Blood Pressure

According to Table 5, no significant between-group differences were reported in any of the six articles assessing systolic or diastolic blood pressure (SBP and DBP respectively). Three studies observed a reduction in the SBP. Specifically, the combination of IC, strength training and liraglutide was effective to reduce SBP but not DBP [15]. On the other hand, the protocols conducted by Tsai, Chan, Liang, Hsu, and Lee [14] and Verrusio, Andreozzi, Renzi, Martinez, Longo, Musumeci and Cacciafesta [13] achieved significant reductions in both SBP and DBP. These interventions consisted of 12 weeks of IC at home [14] and 24 weeks of the combination of IC and diet [13]. These two studies have in common a duration of 12 weeks or more (higher than that from most of the included studies) and a sample comprised by metabolic syndrome patients.

### 3.6. Anthropometric and Body Composition

Body mass (Table 6) was evaluated by nine articles, with no one reporting significant between-group improvements. Significant within group reduction was observed in four studies. The highest reduction was observed in the study by Valle, Mello, Fortes Mde, Dantas, and Mattos [36]. Specifically, the three experimental groups of that article significantly reduced their body mass. Interestingly, the reduction observed in the diet group was larger than that found in the IC group, but lower than the reported in the group that performed exercise and also had diet (close to 10% of the body mass at baseline).

The remaining effective protocols to reduce body mass were (a) 12 weeks of IC in the study by Bianco, Bellafiore, Battaglia, Paoli, Caramazza, Farina, and Palma [6] achieving a reduction of 3.1% (2.2kg), (b) the combination of IC and liraglutide [15], and (c) the combination of IC and walking [12].

In addition to body mass, seven articles also reported the changes in body fat and lean body mass (Table 7). Regarding body fat, all seven articles observed significant within group improvements, while two of them also reported between-group differences. Furthermore, four studies observed significant changes after the interventions. There were significant increases in the studies by Bianco, Bellafiore, Battaglia, Paoli, Caramazza, Farina, and Palma [6]; Kyrolainen, Hackney, Salminen, Repola, Hakkinen, and Haimi [37]; and Petersen, Hastings and Gottschall [16]. On the other hand, the group that carried out a diet intervention (without physical exercise) in the study by Valle, Mello, Fortes Mde, Dantas, and Mattos [36] experienced a significant reduction in the lean body mass.

## 4. Discussion

The main finding of the current systematic review was that IC may be effective for enhancing VO_2max_, HDL, and lean body mass levels, and also for reducing body fat mass, SBP, DBP, LDL, and triglycerides. However, the studies included in the current manuscript often report within-group differences and no between-group differences. In fact, some of the study protocols included a single-group design, so comparison with a control group cannot be done. Furthermore, other studies involving two groups did not compare between IC and other types of physical exercise or usual care, but compared the effects of IC between two different populations (i.e., pathological vs. non-pathological, or premenopausal vs. postmenopausal). Only one of the studies was a randomized controlled trial, thus further high-quality studies are needed in order to increase the quality of the evidence and to enable the meta-analysis calculations.

Regarding aerobic capacity, 2–3 days per week of IC reported improvement between 8–10.5% on VO_2max_ or VO2 [6,33,34,37,38]. Only one study observed an increase lower than 4.8% and its sample was comprised of in women with fibromyalgia [33]. It can be explained by the fact that this group had a lower attendance rate (71%) compared with the matched healthy controls (attendance rate 81%), with a consequent higher improvement (8.5%) [33]. As the intensity, frequency, and duration of exercise is of particular importance for patients with FM, because too much exercise can exacerbate symptoms [39], the training characteristics of the study by Bardal, Roeleveld, and Mork [33] showed that IC can be a recommended exercise in this population [33]. However, an attendance rate of ~70% or more is required to obtain higher improvements [40]. These adherence problems have been previously reported in women with fibromyalgia [41], thus future studies may be focused on the increase of the motivation and the reduction of the dropout rate in this population.

The intensity of IC activities, which involves some high intensity periods and where it has been observed that during recovery, the average VO2 was significantly higher than performing continuous intensity exercise [26]. Thus, IC may have some advantages to increase energy metabolism after cessation of exercise [42], which may be mediated, among others, by an increase in blood lactate concentration [43]. In this regard, the potential role of the excess post exercise oxygen consumption (EPOC) to reduce weight is still controversial [44,45]. For instance, in the study by Abboud, Greer, Campbell, and Panton [45], there were no significant effect of high intensity resistance training on resting metabolic rate in trained men. Thus, further research is needed to reduce the controversy.

Regarding blood pressure and taking into account that hypertension is a risk factor for cardiovascular diseases in subjects with metabolic syndrome, physical training should represent the primary therapeutic approach to prevent these diseases [46]. According to the results observed in the current systematic review, the benefits of IC on reducing blood pressure is higher if the duration of the training is longer [13,14]. A decrease of 4.3% on SBP was observed after 3 months of IC training [14], while the drop on SBP was 11.8% after 6 months [13]. On the other hand, exercise intensity could be a determining factor in modifying the post-exercise hypotension response [47], so IC seems to be a physical activity with adequate intensity, since it has been effective to reduce arterial blood pressure [13,14]. This reduction, in healthy subjects, was observed even at the end of a IC session, where after increasing blood pressure during the session, 30 min after completion of the session it significantly dropped with respect to the initial one (−7.5%), and it remains so until 3 h after the end of that session [48].

In subjects with metabolic syndrome, physical training should represent the first-line therapeutic approach to reduce cardiovascular morbidity and mortality [49], with an emphasis on body composition. In this population, it has been observed that a program that combines IC and diet significantly decreases body mass and cholesterol levels [36]. It must be noted that in that study, the three experimental groups (diet, exercise, and exercise + diet) achieved significant improvements in LDL, fat mass, and body mass. However, the reduction in body mass observed in the diet group involved a significant reduction in the lean body mass. Thus, diet without exercise may be inadequate to successfully improve the body composition. Other studies have evaluated the effects of exercise without diet and observed that body mass decreased but not significantly [8,34]. The reason for this could be the lack of control of diet, and as we mentioned before, the intensity may have not been sufficient to exceed the lactate threshold, which determines the magnitude of the slow component of VO2 [50], causing an increase in energy metabolism after cessation of exercise. This is consistent with previous studies, where the combination of dietary changes associated with exercise was the most effective way for weight loss [51]. Therefore, physical exercise seems to be essential to appropriately manage body composition, since it was the only alternative to reduce body mass and fat mass, without a reduction in the lean body mass. 

Apart from body mass, fat mass and muscle mass, IC may be effective in increasing bone mineral density (BMD) in the arms, legs, pelvis, and spine [16]. These findings can indicate that this type of training might be an effective method of increasing BMD in older and untrained populations. An increase in BMD could effectively decrease the relative risk of a fracture. Specifically, the increase in BMD in the pelvis and legs is particularly important, since the hip is the most common and devastating fracture site for the elderly with osteoporosis [52].

Regarding the lipid profile, the combination of exercise and diet seems to be the most effective way to increase HDL and reduce LDL, total cholesterol and triglycerides levels [13,35]. In the study by Valle, Mello, Fortes Mde, Dantas, and Mattos [36], total cholesterol levels were not reduced in the IC group, which may be caused by the significant increment in HDL levels after the intervention. A meta-analysis of 51 studies with moderate-to-high aerobic intensity, some of them with a dietary intervention, showed a large variability in lipid profile: around 50% of the studies obtained an improvement in HDL and, less frequently, the reduction of triglycerides, total cholesterol, and LDL [53]. Therefore, IC is an effective activity for the improvement of these lipid variables, but diet is also essential in the treatment of dyslipidemia [54,55]. Specifically, a previous study showed the potential benefits of reducing the saturated and trans-fat intake or taking large doses of fish oil and soluble fiber [56]. This may be the reason why, after 3 months of IC, patients with metabolic syndrome showed no changes in lipid profiles [14] along with the fact that high intensity exercise leads to an acceleration of glycogenolysis, with the consequent lower contribution of fat [57]. In sum, IC exercise combined with diet seems to be a very effective method to improve lipid profiles.

The current systematic review has some limitations. Firstly, the search was limited to studies written in Spanish or English, so there could be interesting articles published in other languages that have not been included here. Secondly, most of the studies were not randomized controlled trials. Thus, there were some single-group studies and meta-analysis could not be performed. Therefore, further studies with high-quality design are required in order to improve the quality of the evidence about the effects of IC on health parameters. Finally, there were some variables included in the articles but not reported here because there were less than three articles reporting effects and the extraction of conclusions was not possible.

Despite these limitations, this systematic review provides relevant information about the effects of IC in health-related variables such as blood pressure, body composition, lipid profile, and aerobic capacity.

## 5. Conclusions

Three months of IC, as standalone therapy or combined with strength training or liraglutide, may be effective to improve aerobic capacity. Systolic and diastolic blood pressure can also be reduced especially after 6 months intervention combining IC and diet. Combination of exercise and diet may also be recommended to increase HDL and reduce triglyceride, total cholesterol, and LDL, as well as lose weight without losing muscle mass. Although the observed results are consistent and in line with previous research, the quality of the evidence was very low and further studies are needed.

## Figures and Tables

**Table 1:** Main characteristics of the sample

Study	N		Age	BMI	Group	Exercise	Characteristics
	Male	Female	(years)			Program	
Bardal, 2015	0	16	54.0 ± 7.3	28.1 ± 3.4	EG	IC	Fibromyalgia
	0	19	52.0 ± 8.8	25.7 ± 3.4	CG		Healthy
Bianco, 2010	0	14	22.6 ± 2.1		EG	IC	Overweight
Hedman, 2017	0	21	34.0 ± 7	25.2 ± 3.8	EG	IC	Sedentary
	0	21	35.0 ± 7	23.5 ± 4.2	CG	No	
Kyrolainen, 2018	0	17	27.0 ± 2	25.7 ± 4.6	EG	IC + ST	65% overweight
Lundberg, 2017	0	25	49.1 ± 0.4	24.0 ± 0.5	EG1	IC	Postmenopausal women
	0	24	53.7 ± 0.6	23.7 ± 0.4	EG2		
Mensberg, 2017	10	6	55.6 ± 12	32.4 ± 5.2	EG1	IC + ST + PL	Type 2 diabetes
	13	4	56.5 ± 9	32.5 ± 3.7	EG2	IC + ST + LI	
Petersen, 2017	6	14	47.6 ± 10.3		EG1	IC + BP	Healthy sedentary
					EG2	IC + BB	
Sykes, 2004	0	15	42.9 ± 5.2	24.5 ± 1.5	EG	IC + TR	Premenopausal with overweight
Tsai, 2015	17	16	52.1 ± 10.9	30.4 ± 6.0	EG	IC (H)	Metabolic syndrome or diabetes type 2
Valle, 2010	0	10	24.0 ± 3.2	26.8 ± 2.0	EG1	IC	Healthy
	0	10	23.6 ± 3.9	29.4 ± 3.5	EG2	IC + D	
	0	10	23.5 ± 1.8	27.6 ± 1.5	EG3	D	
	0	10	24.1 ± 3.5	27.5 ± 1.7	CG	No	
Varkey, 2009	3	17	49.0		EG	IC	Patients with migraine
Verrusio, 2016	3	7	62.5 ± 4.7		EG1	D	Metabolic syndrome
	8	2	60.7 ± 6.8		EG2	D + TR + PS	
	6	4	59.2 ± 9.1		EG3	IC + D	
Yoon, 2017	0	12	13.3 ± 0.4	20.1 ± 1.1	EG1	IC	Healthy
	0	12	13.4 ± 0.4	19.3 ± 1.5	EG2	BE	

BMI: body mass index; EG: experimental group; CG: control group; IC: indoor cycling; ST: strength training; PL: placebo; LI: liraglutide; BP: BodyPump^®^; BB: BodyBalance^®^; TR: walking treadmill; H: home; D: diet; PT: physical training; BE: bicycle exercise; MetS: metabolic syndrome.

**Table 2:** Main characteristics of the exercise interventions

Study	Groups	Exercise Program	Duration (Weeks)	Frequency (Days/Week)	Session Time (Min)	Mean Intensity %HRmax RPE	
Bardal, 2015	EG	IC	12	2	45–60	74 ± 6.4	13.7 ± 2
	CG	IC	12	2	45–60	78 ± 8.2	13.6 ± 1.2
Bianco, 2010	EG	IC	12	3	53	152.6 ± 23.1 ^a^	
Hedman, 2017	EG	IC	12	3	45–60		
	CG	No	12				
Kyrolainen, 2018	EG	IC + ST	9	3	30–55	58–91 ^b^	
Lundberg, 2017	EG1	IC	12	3	60	80	
	EG2	IC	12	3	60	80	
Mensberg, 2017	EG1	IC + ST + PL	16	3	60	65–85	
	EG2	IC + ST + LI	16	3	30	65–85	
Petersen, 2017	EG1	IC + BP	24	5–6	60	Program RPM	
	EG2	IC + BB	24	5–6	60	Program RPM	
Sykes, 2004	EG	IC + TR	8	2	80–100		12–13
Tsai, 2015	EG	IC (H)	12	2	50		
Valle, 2010	EG1	IC	12	3	45	55 ± 5–85 ± 5	
	EG2	IC + D	12	3	45	55 ± 5–85 ± 5	
	EG3	D	12	Only diet			
	CG	No	12				
Varkey, 2009	EG	IC	12	3	40		14–16
Verrusio, 2016	EG1	D	24	Only diet			
	EG2	PT + TR + D	24	2	60	≤ 75	
	EG3	IC + D	24	2	45–50	≤ 75	
Yoon, 2017	EG1	IC	16	3	60	45–65	
	EG2	BE	16	3	60	45–65	

HR: heart rate; RPE: rate of perceived exertion; EG: experimental group; CG: control group; IC: indoor cycling; ST: strength training; PL: receiving placebo; LI: receiving liraglutide; BP: BodyPump^®^; BB: BodyBalance^®^; TR: walking treadmill; H: home; D: diet; PT: physical training; BE: bicycle exercise; MetS: metabolic syndrome; ^a^: beats per minute; ^b^: %VO_2max_; RPM: Revolutions Per Minute^®^ program.

**Table 3:** Effects of intervention on aerobic capacity (VO_2max_)

Study	Groups	Pre-Test			Post-Test			Effect (Change)	Within-Group *p*-Value	Between-Group *p*-Value
Bardal, 2015	IC	1.6	±	0.3	1.8	±	0.3	0.1	0.001	NS
	IC(FM)	1.5	±	0.3	1.5	±	0.3	0.1	NS	
Bianco, 2010	IC	37.1	±	4.3	40.2	±	4.6	3.1	<0.001	
Kyrolainen, 2017	IC + ST	NR			NR			8.5%	<0.001	
Lundberg, 2017	IC(PrM)	31.5	±	0.6	34.8	±	0.9	3.3	<0.05	NS
	IC(PoM)	30.4	±	0.9	33.5	±	1.1	3.1	<0.05	
Mensberg, 2017	IC + ST (Pl)	2.5	±	0.7	2.9	±	0.8	0.4	<0.01	NS
	IC + ST (Li)	2.9	±	0.9	3.4	±	1.1	0.5	<0.001	
Varkey, 2008	IC	32.9	±	9.8	36.2	±	8.1	3.3	0.044	

IC: indoor cycling; FM: fibromyalgia group; NS: non-significant; ST: strength training; NR: not reported; PrM: premenopausal group; PoM: postmenopausal group; Pl: placebo group; Li: liraglutide group.

**Table 4:** (**a**) Effects of intervention on serum lipids (triglycerides and total cholesterol). (**b**) Effects of intervention on serum lipids (high-density lipoproteins and low-density lipoproteins).

**(a)**																	
**Study**	**Groups**	**Pre-Test**			**Post-Test**			**Effect (Change)**	***p*-Value**	**Pre-Test**			**Post-Test**			**Effect (Change)**	***p*-Value**
		Triglycerides								Total colesterol							
Kyrolainen, 2017	IC + ST	1.0	±	0.4	1.0	±	0.3	−0.03	NS	4.8	±	0.3	4.4	±	0.7	−0.4	<0.05
Mensberg, 2017	IC + ST (Pl)	1.6	±	1.2	1.4	±	1.1	−0.2	NS	4.2	±	0.9	4.3	±	0.9	0.1	NS
	IC + ST (Li)	1.7	±	1.2	1.5	±	1	−0.2	NS	4.7	±	1.1	4.4	±	1.3	−0.3	NS
Tsai, 2015	IC	1.9	±	1.1	1.8	±	1	−0.1	NS	4.7	±	0.8	4.6	±	1.1	−0.1	NS
Valle, 2010	IC	102.1	±	11.8	97.1	±	11.9	−5	<0.05	179.9	±	11.1	173.1	±	11.5	−6.8	NS
	IC + D	100.4	±	18.4	92.7	±	18.6	−7.7	<0.05	172.4	±	28.1	161.8	±	26.3	−10.6	<0.05
	D	102.6	±	6.7	96.1	±	5.5	−6.5	<0.05	172.9	±	10.9	162.3	±	10.4	−10.6	<0.05
	CG	98.1	±	6.5	98.9	±	5.9	0.8	NS	173.3	±	10.9	175.8	±	11.2	2.5	NS
Verrusio, 2016	IC + D	201	±	152.4	172.8	±	86.7	−28.2	NS	246.3	±	68.5	212.5	±	34.8	−33.8	0.04
	PT + D	160.1	±	8.7	130.7	±	34.3	−29.4	0.001	240	±	30.5	210.6	±	22.5	−29.4	0.001
	D	153.1	±	18.8	153.2	±	96.4	0.1	NS	223.9	±	28.6	220.4	±	31.4	−3.5	NS
Yoon, 2017	IC									163.2	±	11.5	152.4	±	9.0	−10.7	NS
	BE									165.7	±	14.2	158.3	±	12.5	−7.4	NS
**(b)**																	
**Study**	**Groups**	**Pre-Test**			**Post-Test**			**Effect (Change)**	***p*-Value**	**Pre-Test**			**Post-Test**			**Effect (Change)**	***p*-Value**
		High-density lipoproteins								Low-density lipoproteins							
Kyrolainen. 2017	IC + ST	1.4	±	0.3	1.5	±	0.3	0.1	<0.05	2.6	±	0.5	2.5	±	0.5	−0.1	NS
Mensberg. 2017	IC + ST (Pl)	1.2	±	0.3	1.3	±	0.4	0.1	NS	2.2	±	0.9	2.3	±	0.8	0.1	NS
	IC + ST (Li)	1.2	±	0.4	1.2	±	0.3	0	NS	2.6	±	1	2.4	±	1.2	−0.2	NS
Tsai. 2015	IC	1.3	±	0.3	1.3	±	0.3	0	NS	2.8	±	0.7	2.9	±	0.9	0.1	NS
Valle. 2010	IC	40.8	±	2.8	44.1	±	2.2	3.3	<0.05	123.4	±	12.7	114.9	±	13	−8.5	<0.05
	IC + D	41.3	±	3.9	44.6	±	2.7	3.3	<0.05	112.7	±	27.7	103.5	±	27	−9.2	<0.05
	D	42.2	±	2.1	41.4	±	1.8	−0.8	NS	111.7	±	11	105	±	1.1	−6.7	<0.05
	CG	41.2	±	2.6	41.5	±	2.3	0.3	NS	114.8	±	10.7	116.6	±	11	1.8	NS
Verrusio. 2016	IC + D	52.7	±	12.6	52.9	±	11.7	0.2	NS								
	PT + D	53.1	±	12.3	49.9	±	10.5	−3.2	NS								
	D	53.1	±	9.4	50.9	±	11.3	−2.2	NS								
Yoon. 2017	IC									89.3	±	6.8	80.3	±	5.2	−8.92	NS
	BE									90.3	±	8.9	82.8	±	7.4	−7.5	NS

IC: indoor cycling; ST: strength training; NS: nonsignificant; Pl: placebo group; Li: liraglutide group; D: diet group; CG: control group; PT: physical training; BE: bicycle exercise; *p*-value: *p*-value within group. There were no between-group significant differences.

**Table 5:** Effects of intervention on blood pressure

Study	Groups	Pre-Test			Post-Test			Effect (Change)	*p*-Value	Pre-Test			Post-Test			Effect (Change)	*p*-Value
		Systolic Blood Pressure								Diastolic Blood Pressure							
Bardal, 2015	IC	117	±	8.1	113	±	11	−4	NS	68	±	6.6	69	±	7.6	1	NS
	IC (FM)	125	±	14	117	±	14	−8	NS	76	±	10	73	±	7	−3	NS
Bianco, 2010	IC	125.5	±	11.2	121.4	±	8	−4.1	NS	73.7	±	5.7	72.6	±	5.8	−1.1	NS
Hedman, 2017	IC	174	±	17	172	±	16	−2	NS								
	CG	170	±	14	167	±	16	−3	NS								
Mensberg, 2017	IC + ST (Pl)	136.4	±	11	135.8	±	11.6	−0.6	NS	84.1	±	7	81.8	±	8	−2.3	NS
	IC + ST (Li)	136.2	±	8.9	130.8	±	8.8	−5.4	<0.01	82.1	±	7	81.5	±	7.2	−0.6	NS
Tsai, 2015	IC	132.1	±	16.5	126.4	±	16.2	−5.7	0.013	76.9	±	10	74.3	±	8.8	−2.6	0.037
Verrusio, 2016	IC + D	144	±	13.5	127	±	18.9	−17	0.03	88	±	7.5	81.5	±	10.6	−6.5	0.004
	PT + D	140	±	10.5	133	±	9.48	−7	0.001	84	±	7	80	±	6.67	−4	NS
	D	130	±	13.3	130	±	13.3	0	NS	82.5	±	4.2	82.5	±	4.24	0	NS

IC: indoor cycling; FM: fibromyalgia group; NS: non-significant; CG: control group; ST: strength training; Pl: placebo group; Li: liraglutide group; D: diet; PT: physical training; *p*-value: *p*-value within group; There were no between-group significant differences.

**Table 6:** Effects of intervention on body mass

Study	Groups	Pre-Test			Post-Test			Effect (Change)	Within-Group *p*-Value	Between-Group *p*-Value
		Body Mass								
Bianco, 2010	IC	70.8	±	8.8	68.6	±	9.2	−2.2	0.0001	
Hedman, 2017	IC	NR	±		NR	±		−0.7	NS	NS
	CG	NR	±		NR	±		0.3	NS	
Kyrolainen, 2017	IC + ST	72.3	±	13	72	±	13	−0.3	NS	
Lundberg, 2017	IC (PrM)	67.6	±	1.4	67.1	±	1.4	−0.5	NS	NS
	IC (PoM)	66.6	±	1.6	65.8	±	1.5	−0.8	NS	
Mensberg, 2017	IC + ST (Pl)	96.8	±	17	95.2	±	18	−1.6	NS	NS
	IC + ST (Li)	101	±	15	97.6	±	15	−3.4	<0.001	
Petersen, 2017	IC + BP	NR	±		NR	±			NS	NS
	IC + BB	NR	±		NR	±			NS	
Sykes, 2004	IC + TR5	62.7	±	4.8	60.7	±	4.9	−2	<0.05	NS
	IC + TR2	63.7	±	6.9	61.8	±	6.8	−1.9	<0.05	
Valle, 2010	IC	68.8	±	7.1	64.9	±	6.6	−3.9	<0.05	NS
	IC + D	74.4	±	8.3	67.1	±	8.9	−7.3	<0.05	
	D	71.4	±	4.2	65.4	±	4.4	−6.0	<0.05	
	CG	71.9	±	6	72.6	±	6.3	0.8	NS	
Yoon, 2017	IC	49.4	±	3.4	49.2	±	3.3	−0.2	NS	NS
	BE	47.5	±	5.7	48.9	±	5.7	1.4	NS	

IC: indoor cycling; CG: control group; NS: non-significant; ST: strength training; PrM: premenopausal group; PoM: postmenopausal group; Pl: placebo group; Li: liraglutide group; BP: BodyPump*^®^*; BB: BodyBalance*^®^*; TR5: walking treadmill spending 2000 calories spread over 5 days; TR2: walking treadmill spending 2000 calories spread over 2 days; D: diet; CG: control group; BE: bicycle exercise.

**Table 7:** Effects of intervention on percentage of body fat and lean body mass

Study	Groups	Pre-Test			Post-Test			Effect (Change)	*p*-Value	Pre-Test			Post-Test			Effect (Change)	*p*-Value
		Percentage of body fat								Lean body mass							
Bianco, 2010	IC	34.9	±	5.5	33.2	±	5.3	−1.7	0.0001	65.1	±	5.5	66.8	±	5.3	1.7	0.0001
Kyrolainen, 2017	IC + ST	32.8	±	8.6	32	±	8.5	−0.8	0.03	26.4	±	9.8	26.6	±	3.4	0.2	0.03
Mensberg, 2017	IC + ST (Pl)	37	±	6.5	34.8	±	7	−2.2	<0.001	58	±	12	58.7	±	12	0.7	NS
	IC + ST (Li)	34.3	±	6.3	31.8	±	6.8	−2.5	<0.001	63.3	±	12	63.4	±	13	0.1	NS
Petersen, 2017	IC + BP	NR	±		NR	±		decreased	0.002	NR	±		NR	±		increased	<0.05
	IC + BB	NR	±		NR	±		decreased	0.019	NR	±		NR	±		increased	<0.05
Sykes, 2004	IC + TR5	30.2	±	4.4	28.8	±	4.4	−1.4	<0.05	43.8	±	4.6	43.2	±	4.6	−0.6	NS
	IC + TR2	29.4	±	2.5	28.2	±	2.3	−1.2	<0.05	44.9	±	2.8	44.3	±	2.6	−0.6	NS
Valle, 2010	IC	32.9	±	2.3	28.5	±	2.3	−4.4	<0.05	46.1	±	3.8	46.3	±	3.6	0.2	NS
	IC + D	33.9	±	5.4	26.7	±	6	−7.2	<0.05	49.0	±	5.5	48.8	±	4.9	−0.2	NS
	D	33.1	±	3.7	30.3	±	3.5	−2.8	<0.05	47.7	±	1.7	45.4	±	1.6	−2.2	<0.05
	CG	31.7	±	3.2	32.1	±	3.1	0.4	<0.05	49.2	±	3.3	49.3	±	3.2	0.1	NS
Yoon, 2017	IC	22.1	±	1.2	20.9	±	1.1	−1.2	<0.05								
	BE	22.4	±	2.8	22.4	±	2.6	−0.1	NS								

IC: indoor cycling; ST: strength training; Pl: placebo group; Li: liraglutide group; NS: nonsignificant; BP: BodyPump*^®^*; BB: BodyBalance*^®^*; NR: no reported; TR5: walking treadmill spending 2000 calories spread over 5 days; TR2: walking treadmill spending 2000 calories spread over 2 days; D: diet; CG: control group; BE: bicycle exercise; *p*-value: *p*-value within-group; There were between-group significant differences in Valle 2010 *p* < 0.05 between groups IC + D and CG, and Yoon 2017 *p* < 0.05 between groups IC and BE.
